# Relationship between site procedural volumes and outcomes of left bundle branch area pacing using stylet-driven leads from the INSIGHT-LBBA Registry

**DOI:** 10.1016/j.hroo.2026.07.011

**Published:** 2026-07-20

**Authors:** Ilya Y. Shadrin, Hillary Mulder, Brooke Alhanti, Nicholas Wold, Gaurav A. Upadhyay, Christopher A. Latanich, Seth Goldbarg, Kevin M. Trulock, Apoor Patel, Zak Loring, Myat Shwe Yee Tun, Adi Lador, Jonathan Na, Paul W. Jones, Stephen B. Ruble, Daniel J. Friedman

**Affiliations:** 1 Duke University Hospital, Durham, North Carolina; 2 Duke Clinical Research Institute, Durham, North Carolina; 3 Boston Scientific, St. Paul, Minnesota; 4 University of Chicago Medicine, Chicago, Illinois; 5 Essentia Health, Duluth, Minnesota; 6 New York Presbyterian Queens, Flushing, New York; 7 Community Heart and Vascular Hospital, Indianapolis, Indiana; 8 Houston Methodist Hospital, Houston, Texas

**Keywords:** Left bundle branch area pacing, Conduction system pacing, Learning curve, Stylet-driven lead, Procedural success

## Abstract

**Background:**

Left bundle branch (LBB) area pacing (LBBAP) is increasingly used for patients with a ventricular pacing indication. Multi-center studies analyzing predominantly lumenless leads have suggested a learning curve for LBBAP; however, analogous data on stylet-driven leads (SDLs) are lacking.

**Objective:**

To assess the relationship between site volume and procedural outcomes and complications using the INGEVITY+ SDL for LBBAP.

**Methods:**

A secondary analysis of the INSIGHT-LBBA multi-site registry was performed, with retrospective data collected across 8 United States sites performing LBBAP with INGEVITY+ SDLs between 2020 and 2023. Outcomes included LBBAP and LBB capture success rates, pacing parameters, fluoroscopy time, number of leads used, and lead-related complications. Outcomes were assessed using a generalized estimated equation based on consecutive implants. A sensitivity analysis was performed restricted to only single- and dual-chamber LBBAP implants.

**Results:**

A total of 1374 patients were enrolled in the primary INSIGHT-LBBA registry. Overall LBBAP success and LBB capture success rates were 94.5% and 78.1%, respectively. LBBAP success rate improved with experience (odds ratio, 1.09; 95% confidence interval, 1.03–1.14, per every 10 successive LBBAP implants; *P* = .001), with similar results in the sensitivity analysis. Fluoroscopy times and complication rates remained low (10.0 ± 9.2 minutes and 2.7%, respectively).

**Conclusion:**

In this secondary analysis of INSIGHT-LBBA using INGEVITY+ SDLs, LBBAP and LBB capture success rates were high and improved with consecutive implants, indicative of a learning curve for implantation. Fluoroscopy times and complication rates remained low without significant change during consecutive implants. These data add to the growing literature supporting safe and effective use of SDLs for LBBAP.

**Key Findings**

▪ In a secondary analysis of the INSIGHT-LBBA registry, the success rate of both left bundle branch area pacing and left bundle branch capture was high and improved with consecutive cases, indicative of a learning curve.
▪ Overall lead-related complications remained low at 2.7% by 1 year.
▪ No evidence of learning curve bias for lead-related complications or procedural times, with no outcomes differences based on prior experience with lumenless leads.

## Introduction

Since its inception in 2017,^1^ left bundle branch (LBB) area pacing (LBBAP) has rapidly become a preferred pacing strategy for patients with a ventricular pacing indication. Consistent with most novel technologies, a multicenter European registry of LBBAP implants (MELOS^2^) recently identified a learning curve for LBBAP implantation across 14 different sites. Because a majority (83.8%) of these procedures involved the use of a lumenless lead, analogous performance data for stylet-driven leads (SDLs) are relatively lacking. To this end, a multicenter registry (INSIGHT-LBBA^3^) recently assessed the safety and efficacy of an active fixation SDL (INGEVITY+) during consecutive LBBAP attempts across 8 United States high-volume centers. All pre-specified performance goals for pacing, sensing, and lead-related complications (LRC) were met, which subsequently contributed to the Food and Drug Administration approval for the use of the INGEVITY+ lead for LBBAP.^4^^,^^5^ Given the multi-site nature of the initial registry, we sought to address the learning curve knowledge gap surrounding SDLs. To this end, we performed a secondary analysis of the INSIGHT-LBBA registry assessing the relationship between site volume and procedural outcomes and complications using the INGEVITY+ lead for LBBAP.

## Methods

### Study design

The primary INSIGHT-LBBA study was a retrospective, multi-center, investigator-initiated non-randomized registry (NCT 06014866) funded by Boston Scientific (Marlborough, MA) that included patients with attempted LBBAP implantation for a bradycardia indication between Q3 of 2020 and Q3 of 2023. The Duke Clinical Research Institute served as the coordinating center for the study. All LBBAP implants collected for the primary registry were performed using the Boston Scientific INGEVITY+ lead, a 59 cm 6 Fr active fixation SDL delivered through 1 of 4 site-specific pacing catheters (SSPC 1–4).

Data from enrolled patients were collected retrospectively on consecutive implants at each participating center, and organized via a custom REDCap electronic database to maximize data uniformity and completeness. As all entered data was de-identified with no collection of source documents, each site was exempt from requiring patient consent by the local institutional review board. All patients were required to have at least 90 days of follow-up to be included in the study. Participating sites were requested to submit supplementary data collection voluntarily, which included additional procedural information not obtained in the initial data registry. The research reported in this paper adhered to guidelines for human subject research as outlined in the Helsinki Declaration.

### Outcomes

Procedural success was measured in 2 ways—LBBAP success rate and LBB capture success rate. LBBAP success was defined as either capture of left interventricular septal tissue or capture of the left-sided conduction system (LBB capture), which was further defined based on MELOS criteria described previously.^2^^,^^3^ Briefly, LBB capture was confirmed when 1 of the following criteria were met: (1) QRS morphology change during either threshold testing or programmed electrical stimulation; (2) onset of pacing stimulus to R-wave peak time of lead V_6_ (V_6_-RWPT) < 80 ms (if narrow QRS or isolated right bundle branch block) or <90 ms (if more advanced ventricular conduction disease such as bifascicular block, LBB block, or complete heart block with wide escape rhythm); (3) LBB potential to V_6_-RWPT interval within 10 ms of pacing stimulus to V_6_-RWPT; or (4) V_6_–V_1_ interpeak interval (peak of R-wave in V_6_ to peak of terminal R-wave in V_1_) of >40 ms. Capture of left septal tissue was defined as the presence of a terminal R/r in lead V_1_ without meeting any LBB capture criteria.

Measured intraprocedural parameters included number of leads used per implant, and total procedure and fluoroscopy time. Pacing lead parameters at implant and 3 months (supplemented with 6-month data if not available at 3 months) included sensing (mV), impedance (Ω), and pacing threshold (V). Peri- and post-procedural complications included any LRC or lead dislodgement, as described in the initial INSIGHT-LBBA registry.^3^

### Statistical analysis

Initial secondary analysis of INSIGHT-LBBA data was performed on all enrolled patients with non-missing device type, with a sensitivity analysis restricted to those patients who received either a single or dual-chamber pacemaker. For descriptive purposes, consecutive implants at each site were grouped into 4 categories—implants #1–25, #26–50, #51–100, and #101+. Missing data were excluded from denominators for descriptive analyses, and modeling was performed using a complete case approach. Baseline characteristics included demographics, medications at enrollment, comorbidities, pacing indication, heart failure status, and echocardiographic and electrocardiogram (ECG) parameters.

A generalized estimating equation model was used to evaluate the associations between implant order and either LBBAP success, LBB capture success, intraprocedural outcomes, or pacing parameters, taking note of clustering of patients within sites and using an independent working correlation matrix. The linearity assumption for implant order with respect to outcomes was evaluated using natural cubic splines. If major violations were found, 2 piecewise linear splines were created for inclusion in the model. For LBBAP success, LBB capture success, and leads per implant (1 vs 2 or 3 leads), a binomial distribution and logit link were used, whereas a normal distribution with identity link was used for the intraprocedural outcomes and pacing parameters. Results of the logistic regression models were presented as counts (%) within levels of the categorized implant order and odds ratios (OR) (95% confidence interval [CI]) per a 10-unit increase in continuous implant order. Results of the linear regression models were presented as mean (standard deviation) within levels of categorized implant order and mean difference (95% CI) in outcome per a 10-unit increase in continuous implant order. Modeling was performed both unadjusted and adjusted for age, sex, heart failure type, and device type (pacemaker vs other).

To evaluate the association between implant order and procedural complications, a Cox proportional hazards model was used. The proportional hazards assumption was checked using weighted Schoenfeld residuals, with no major violations noted. To account for clustering, a robust sandwich estimator was used to calculate standard errors. Results were presented using Kaplan–Meier event rates at 12 and 24 months, and Kaplan–Meier curves truncated at 24 months, as well as hazard ratios (HRs) (95% CI) per a 10-unit increase in continuous implant order. Outcomes were evaluated in both unadjusted and adjusted models.

## Results

### Baseline characteristics

A total of 1374 patients were enrolled in INSIGHT-LBBA with no missing data. Baseline characteristics demonstrated a mean age of 75.1 ± 10.6 years, 58.5% male sex, 81.1% Caucasian, mean ejection fraction (EF) of 54.7% ± 11.1% (19.2% with heart failure with reduced EF [HFrEF]), and a pacing indication of either high-grade atrioventricular block (47.4%) or sinus node dysfunction (36.0%) in a majority of patients (Table 1). Average baseline QRS duration pre-LBBAP implant was 117.7 ± 30.6 ms if intrinsic and 163.2 ± 29.5 ms if paced (Table 1). Relative to earlier LBBAP implants (1–25), later implants (101+) involved patients with a higher prevalence of idiopathic cardiomyopathy (26% vs 8.2%), HFrEF (27.3% vs 11.9%), and implantation of either a CRT-P or CRT-D (20% vs 5.1%) due to a higher number of patients meeting class I or II criteria for CRT (21.5% vs 2.6%, Table 1). A total of 1122 patients received either a single- or a dual-chamber pacemaker and were included in the sensitivity analysis, with similar baseline characteristics to the overall cohort (Table 2). In this cohort, no clinically significant baseline differences were observed when stratified by implant category.

**Table 1:** Baseline patient characteristics by site implant order in the overall cohort

Characteristic	Overall (N = 1374)	Implants 1–25 (N = 194)	Implants 26–50 (N = 173)	Implants 51–100 (N = 281)	Implants 101+ (N = 726)
Number of sites		8	7	7	5
Age (years)					
N	1363	194	173	280	716
Mean (SD)	75.1 (10.6)	76.3 (9.8)	76.1 (10.2)	75.8 (10.9)	74.3 (10.7)
Median [Q1, Q3]	76.0 (69.0, 82.0)	78.0 (69.0, 83.0)	77.0 (70.6, 83.0)	77.0 (69.3, 83.4)	75.6 (69.0, 81.1)
Male	804 (58.5%)	114 (58.8%)	103 (59.5%)	158 (56.2%)	429 (59.1%)
Device type					
CRT-D	69 (5.0%)	3 (1.5%)	2 (1.2%)	5 (1.8%)	59 (8.1%)
CRT-P	100 (7.3%)	7 (3.6%)	1 (0.6%)	6 (2.1%)	86 (11.8%)
Dual ICD	5 (0.4%)	0 (0.0%)	1 (0.6%)	1 (0.4%)	3 (0.4%)
Dual PM	1146 (83.4%)	178 (91.8%)	163 (94.2%)	264 (94.0%)	541 (74.5%)
Leadless PM	1 (0.1%)	0 (0.0%)	1 (0.6%)	0 (0.0%)	0 (0.0%)
Other - Bi-V	2 (0.1%)	1 (0.5%)	0 (0.0%)	0 (0.0%)	1 (0.1%)
Single PM	51 (3.7%)	5 (2.6%)	5 (2.9%)	5 (1.8%)	36 (5.0%)
Leads used per implant					
1	1242 (90.4%)	182 (93.8%)	162 (93.6%)	269 (95.7%)	629 (86.6%)
2	106 (7.7%)	11 (5.7%)	11 (6.4%)	11 (3.9%)	73 (10.1%)
3	25 (1.8%)	1 (0.5%)	0 (0.0%)	1 (0.4%)	23 (3.2%)
4	1 (0.1%)	0 (0.0%)	0 (0.0%)	0 (0.0%)	1 (0.1%)
Race∗					
American Indian or Alaska Native	8 (0.6%)	2 (1.0%)	1 (0.6%)	1 (0.4%)	4 (0.6%)
Asian	77 (5.6%)	12 (6.2%)	13 (7.5%)	37 (13.2%)	15 (2.1%)
Black/African American	128 (9.3%)	31 (16.0%)	28 (16.2%)	26 (9.3%)	43 (5.9%)
Native Hawaiian or Other Pacific Islander	1 (0.1%)	1 (0.5%)	0 (0.0%)	0 (0.0%)	0 (0.0%)
White/Caucasian	1115 (81.1%)	142 (73.2%)	123 (71.1%)	203 (72.2%)	647 (89.1%)
Other/Unknown	42 (3.1%)	6 (3.1%)	7 (4.0%)	15 (5.3%)	14 (1.9%)
Ethnicity					
Hispanic or Latino	35 (2.6%)	5 (2.6%)	7 (4.2%)	17 (6.1%)	6 (0.8%)
Not Hispanic or Latino	1315 (97.2%)	186 (96.9%)	161 (95.8%)	261 (93.5%)	707 (99.0%)
Unknown	3 (0.2%)	1 (0.5%)	0 (0.0%)	1 (0.4%)	1 (0.1%)
**Medications at Enrollment**					
ACE Inhibitor	320 (25.0%)	38 (21.2%)	36 (24.2%)	57 (24.7%)	189 (26.3%)
Angiotensin receptor blocker (ARB)	298 (23.3%)	45 (25.1%)	34 (22.8%)	50 (21.6%)	169 (23.5%)
Angiotensin receptor-Neprilysin inhibitor (ARNi)	25 (2.0%)	1 (0.6%)	2 (1.3%)	2 (0.9%)	20 (2.8%)
Flecainide	22 (1.7%)	3 (1.7%)	4 (2.7%)	7 (3.0%)	8 (1.1%)
Propafenone	0 (0.0%)	0 (0.0%)	0 (0.0%)	0 (0.0%)	0 (0.0%)
Other antiarrhythmics	153 (12.0%)	18 (10.1%)	16 (10.7%)	21 (9.1%)	98 (13.6%)
Anticoagulant	583 (45.6%)	88 (49.2%)	68 (45.6%)	93 (40.3%)	334 (46.5%)
Diuretics	560 (43.8%)	76 (42.5%)	62 (41.6%)	81 (35.1%)	341 (47.4%)
Beta blockers	608 (47.6%)	79 (44.1%)	67 (45.0%)	103 (44.6%)	359 (49.9%)
Sodium-glucose cotransporter-2 (SGLT2i)	100 (7.8%)	14 (7.8%)	6 (4.0%)	12 (5.2%)	68 (9.5%)
Aldosterone antagonists	111 (8.7%)	18 (10.1%)	5 (3.4%)	7 (3.0%)	81 (11.3%)
Calcium channel blockers	383 (30.0%)	49 (27.4%)	51 (34.2%)	84 (36.4%)	199 (27.7%)
Clinical heart failure diagnosis	614 (44.7%)	71 (36.6%)	70 (40.5%)	99 (35.2%)	374 (51.5%)
Ischemic heart disease	136 (9.9%)	20 (10.3%)	13 (7.5%)	19 (6.8%)	84 (11.6%)
Idiopathic cardiomyopathy	241 (17.5%)	16 (8.2%)	21 (12.1%)	15 (5.3%)	189 (26.0%)
Valvular heart disease	102 (7.4%)	18 (9.3%)	14 (8.1%)	19 (6.8%)	51 (7.0%)
Hypertrophic cardiomyopathy	19 (1.4%)	3 (1.5%)	2 (1.2%)	5 (1.8%)	9 (1.2%)
Congenital heart disease	4 (0.3%)	1 (0.5%)	1 (0.6%)	1 (0.4%)	1 (0.1%)
Left ventricular hypertrophy	71 (5.2%)	11 (5.7%)	4 (2.3%)	10 (3.6%)	46 (6.3%)
Infiltrative cardiomyopathy	7 (0.5%)	3 (1.5%)	2 (1.2%)	1 (0.4%)	1 (0.1%)
No disease (age-related)	92 (6.7%)	4 (2.1%)	6 (3.5%)	23 (8.2%)	59 (8.1%)
HF type					
Heart failure with preserved Ejection fraction (HFpEF)	337 (24.5%)	48 (24.7%)	49 (28.3%)	68 (24.2%)	172 (23.7%)
Heart failure with reduced ejection Fraction (HFrEF)	264 (19.2%)	23 (11.9%)	18 (10.4%)	25 (8.9%)	198 (27.3%)
**Cardiac Assessment**					
QRS width pre-LBBAP implant (intrinsic)					
N	1241	178	154	263	646
Mean (SD)	117.7 (30.6)	112.1 (36.6)	112.1 (28.9)	115.5 (27.7)	121.6 (29.9)
Median [Q1, Q3]	112 (92.0, 142)	105 (90.0, 138)	104 (88.0, 134)	112 (92.0, 138)	118 (96.0, 146)
QRS width pre-LBBAP implant (paced)					
N	86	8	7	6	65
Mean (SD)	163.2 (29.5)	141.1 (53.9)	152.3 (34.6)	152.0 (11.9)	168.2 (24.6)
Median [Q1, Q3]	164 (148, 182)	138 (102, 176)	160 (131, 166)	154 (148, 160)	168 (152, 186)
QRS width post-LBBAP implant (paced)					
N	824	94	98	139	493
Mean (SD)	122.6 (25.1)	125.9 (42.6)	126.3 (23.0)	121.4 (17.5)	121.6 (22.7)
Median [Q1, Q3]	121 (108, 134)	122 (104, 140)	125 (112, 140)	122 (110, 132)	118 (108, 133)
Cardiac surgeries within 30 days of implant	109 (7.9%)	11 (5.7%)	12 (6.9%)	25 (8.9%)	61 (8.4%)
LVEF					
N	1274	166	149	251	708
Mean (SD)	54.7 (11.1)	57.9 (9.6)	57.5 (8.5)	57.1 (8.7)	52.5 (12.2)
Median [Q1, Q3]	57.0 (53.0, 63.0)	59.0 (55.0, 63.0)	58.0 (55.0, 63.0)	57.0 (55.0, 63.0)	55.0 (48.0, 58.5)
Septal wall thickness (mm)					
N	1117	136	125	207	649
Mean (SD)	11.6 (2.7)	11.7 (2.8)	11.7 (2.5)	11.5 (3.0)	11.5 (2.5)
Median [Q1, Q3]	11.0 (10.0, 13.0)	11.0 (10.0, 13.0)	11.0 (10.0, 13.0)	11.0 (9.9, 13.0)	11.0 (10.0, 13.0)
BMI					
N	1342	190	164	264	724
Mean (SD)	29.3 (6.6)	29.8 (7.2)	29.0 (6.7)	28.7 (6.2)	29.5 (6.5)
Median [Q1, Q3]	28.5 (24.6, 33.0)	28.5 (24.8, 34.4)	27.7 (24.7, 31.5)	28.5 (23.8, 32.4)	28.7 (24.8, 33.1)
Pacing indications∗					
Sinus node dysfunction	495 (36.0%)	87 (44.8%)	86 (49.7%)	126 (44.8%)	196 (27.0%)
Atrioventricular block	651 (47.4%)	94 (48.5%)	77 (44.5%)	106 (37.7%)	374 (51.5%)
Neurocardiogenic syncope	6 (0.4%)	1 (0.5%)	0 (0.0%)	1 (0.4%)	4 (0.6%)
Meets class I or II criteria for CRT	168 (12.2%)	5 (2.6%)	3 (1.7%)	4 (1.4%)	156 (21.5%)
Ablate and pace procedure	114 (8.3%)	9 (4.6%)	7 (4.0%)	14 (5.0%)	84 (11.6%)
Other	226 (16.4%)	37 (19.1%)	35 (20.2%)	67 (23.8%)	87 (12.0%)
**Associated diseases and risk factors**					
Hypertension	1170 (85.2%)	162 (83.5%)	152 (87.9%)	251 (89.3%)	605 (83.3%)
Diabetes	537 (39.1%)	63 (32.5%)	59 (34.1%)	108 (38.4%)	307 (42.3%)
Renal disease	404 (29.4%)	48 (24.7%)	51 (29.5%)	65 (23.1%)	240 (33.1%)
Chronic pulmonary disease	332 (24.2%)	32 (16.5%)	29 (16.8%)	51 (18.1%)	220 (30.3%)
Peripheral arterial disease	141 (10.3%)	25 (12.9%)	10 (5.8%)	21 (7.5%)	85 (11.7%)
Other	560 (40.8%)	76 (39.2%)	69 (39.9%)	109 (38.8%)	306 (42.1%)
No associated diseases and risk factors	86 (6.3%)	15 (7.7%)	8 (4.6%)	13 (4.6%)	50 (6.9%)

ACE = angiotensin-converting enzyme; ARB = angiotensin receptor blocker; ARNi = angiotensin receptor–neprilysin inhibitor; Bi-V = biventricular; BMI = body mass index; CRT = cardiac resynchronization therapy; CRT-D = cardiac resynchronization therapy defibrillator; CRT-P = cardiac resynchronization therapy pacemaker; HF = heart failure; HFpEF = heart failure with preserved ejection fraction; HFrEF = heart failure with reduced ejection fraction; ICD = implantable cardioverter-defibrillator; LBBAP = left bundle branch area pacing; LVEF = left ventricular ejection fraction; Q1 = first quartile; Q3 = third quartile; SD = standard deviation; SGLT2i = sodium-glucose cotransporter-2 inhibitor.

∗ Categories are not mutually exclusive.

**Table 2:** Baseline patient characteristics by site implant order in the sensitivity cohort

Characteristic	Overall (N = 1122)	Implants 1–25 (N = 178)	Implants 26–50 (N = 167)	Implants 51–100 (N = 264)	Implants 101+ (N = 513)
Number of sites		8	7	7	5
Age (years)					
N	1114	178	167	263	506
Mean (SD)	75.8 (10.0)	76.3 (9.7)	76.4 (10.1)	76.4 (10.2)	75.1 (9.9)
Median [Q1, Q3]	76.9 (70.0, 82.0)	77.6 (69.0, 83.0)	77.0 (71.0, 83.0)	77.0 (70.0, 84.0)	76.0 (70.0, 81.6)
Male	643 (57.3%)	104 (58.4%)	99 (59.3%)	151 (57.2%)	289 (56.3%)
Device type					
Dual PM	1073 (95.6%)	174 (97.8%)	162 (97.0%)	259 (98.1%)	478 (93.2%)
Single PM	49 (4.4%)	4 (2.2%)	5 (3.0%)	5 (1.9%)	35 (6.8%)
Leads used per implant					
1	1050 (93.6%)	167 (93.8%)	157 (94.0%)	253 (95.8%)	473 (92.2%)
2	59 (5.3%)	10 (5.6%)	10 (6.0%)	10 (3.8%)	29 (5.7%)
3	13 (1.2%)	1 (0.6%)	0 (0.0%)	1 (0.4%)	11 (2.1%)
Race∗					
American Indian or Alaska Native	8 (0.7%)	2 (1.1%)	1 (0.6%)	1 (0.4%)	4 (0.8%)
Asian	75 (6.7%)	12 (6.7%)	13 (7.8%)	36 (13.6%)	14 (2.7%)
Black/African American	118 (10.5%)	30 (16.9%)	26 (15.6%)	24 (9.1%)	38 (7.4%)
Native Hawaiian or Other Pacific Islander	1 (0.1%)	1 (0.6%)	0 (0.0%)	0 (0.0%)	0 (0.0%)
White/Caucasian	881 (78.5%)	127 (71.3%)	119 (71.3%)	188 (71.2%)	447 (87.1%)
Other/Unknown	35 (3.1%)	6 (3.4%)	7 (4.2%)	15 (5.7%)	7 (1.4%)
Ethnicity					
Hispanic or Latino	33 (3.0%)	5 (2.8%)	7 (4.3%)	17 (6.5%)	4 (0.8%)
Not Hispanic or Latino	1068 (96.7%)	170 (96.6%)	155 (95.7%)	244 (93.1%)	499 (99.0%)
Unknown	3 (0.3%)	1 (0.6%)	0 (0.0%)	1 (0.4%)	1 (0.2%)
**Medications at Enrollment**					
ACE Inhibitor	251 (24.4%)	34 (20.9%)	35 (24.5%)	55 (25.6%)	127 (25.1%)
Angiotensin Receptor Blocker (ARB)	238 (23.2%)	43 (26.4%)	32 (22.4%)	49 (22.8%)	114 (22.5%)
Angiotensin Receptor-Neprilysin Inhibitor (ARNi)	5 (0.5%)	0 (0.0%)	1 (0.7%)	1 (0.5%)	3 (0.6%)
Flecainide	20 (1.9%)	3 (1.8%)	4 (2.8%)	5 (2.3%)	8 (1.6%)
Propafenone	0 (0.0%)	0 (0.0%)	0 (0.0%)	0 (0.0%)	0 (0.0%)
Other Antiarrhythmics	105 (10.2%)	15 (9.2%)	15 (10.5%)	20 (9.3%)	55 (10.9%)
Anticoagulant	432 (42.1%)	78 (47.9%)	66 (46.2%)	87 (40.5%)	201 (39.7%)
Diuretics	406 (39.5%)	69 (42.3%)	58 (40.6%)	73 (34.0%)	206 (40.7%)
Beta Blockers	436 (42.5%)	70 (42.9%)	63 (44.1%)	93 (43.3%)	210 (41.5%)
Sodium-glucose Cotransporter-2 (SGLT2i)	56 (5.5%)	9 (5.5%)	6 (4.2%)	9 (4.2%)	32 (6.3%)
Aldosterone Antagonists	56 (5.5%)	12 (7.4%)	5 (3.5%)	5 (2.3%)	34 (6.7%)
Calcium Channel Blockers	334 (32.5%)	45 (27.6%)	50 (35.0%)	82 (38.1%)	157 (31.0%)
Clinical heart failure diagnosis	414 (36.9%)	59 (33.1%)	66 (39.5%)	86 (32.6%)	203 (39.6%)
Ischemic heart disease	85 (7.6%)	17 (9.6%)	12 (7.2%)	16 (6.1%)	40 (7.8%)
Idiopathic cardiomyopathy	101 (9.0%)	8 (4.5%)	20 (12.0%)	10 (3.8%)	63 (12.3%)
Valvular heart disease	87 (7.8%)	16 (9.0%)	12 (7.2%)	18 (6.8%)	41 (8.0%)
Hypertrophic cardiomyopathy	11 (1.0%)	2 (1.1%)	2 (1.2%)	4 (1.5%)	3 (0.6%)
Congenital heart disease	3 (0.3%)	1 (0.6%)	1 (0.6%)	0 (0.0%)	1 (0.2%)
Left ventricular hypertrophy	62 (5.5%)	11 (6.2%)	4 (2.4%)	9 (3.4%)	38 (7.4%)
Infiltrative cardiomyopathy	7 (0.6%)	3 (1.7%)	2 (1.2%)	1 (0.4%)	1 (0.2%)
No disease (age-related)	89 (7.9%)	4 (2.2%)	6 (3.6%)	23 (8.7%)	56 (10.9%)
HF type					
Heart failure with preserved Ejection fraction (HFpEF)	309 (27.5%)	45 (25.3%)	49 (29.3%)	62 (23.5%)	153 (29.8%)
Heart failure with reduced ejection Fraction (HFrEF)	96 (8.6%)	14 (7.9%)	14 (8.4%)	19 (7.2%)	49 (9.6%)
**Cardiac assessment**					
QRS width pre-LBBAP implant (intrinsic)					
N	1057	169	148	249	491
Mean (SD)	116.2 (29.2)	112.0 (34.1)	111.5 (28.6)	114.7 (27.1)	119.9 (28.2)
Median [Q1, Q3]	111 (92.0, 140)	103 (90.0, 136)	103 (88.0, 134)	110 (90.0, 138)	116 (96.0, 143)
QRS width pre-LBBAP implant (paced)					
N	29	2	7	4	16
Mean (SD)	156.3 (28.2)	179.5 (72.8)	152.3 (34.6)	158.0 (6.9)	154.8 (23.6)
Median [Q1, Q3]	160 (145, 165)	180 (128, 231)	160 (131, 166)	160 (154, 162)	160 (141, 166)
QRS width post-LBBAP implant (paced)					
N	617	83	96	129	309
Mean (SD)	123.0 (25.1)	125.4 (44.6)	125.9 (23.0)	121.1 (17.5)	122.2 (20.8)
Median [Q1, Q3]	122 (108, 134)	122 (102, 140)	124 (112, 139)	122 (110, 132)	121 (108, 134)
Cardiac surgeries within 30 days of implant	94 (8.4%)	11 (6.2%)	12 (7.2%)	23 (8.7%)	48 (9.4%)
LVEF					
N	1028	152	144	236	496
Mean (SD)	57.7 (7.7)	59.0 (8.6)	57.9 (8.1)	57.7 (7.6)	57.2 (7.2)
Median [Q1, Q3]	57.0 (55.0, 63.0)	60.0 (55.0, 64.5)	58.0 (55.0, 63.0)	57.0 (55.0, 63.0)	57.0 (55.0, 63.0)
Septal wall thickness (mm)					
N	896	125	120	196	455
Mean (SD)	11.6 (2.7)	11.7 (2.9)	11.6 (2.5)	11.5 (3.0)	11.6 (2.6)
Median [Q1, Q3]	11.0 (10.0, 13.0)	11.0 (10.0, 13.0)	11.0 (9.9, 13.0)	11.0 (10.0, 13.0)	11.0 (10.0, 13.0)
BMI					
N	1091	174	158	248	511
Mean (SD)	29.3 (6.6)	30.0 (7.3)	29.0 (6.8)	28.8 (6.2)	29.4 (6.4)
Median [Q1, Q3]	28.3 (24.6, 33.0)	28.7 (24.8, 34.4)	27.6 (24.7, 31.4)	28.6 (23.9, 32.7)	28.5 (24.8, 33.1)
Pacing indications∗					
Sinus node dysfunction	448 (39.9%)	84 (47.2%)	86 (51.5%)	122 (46.2%)	156 (30.4%)
Atrioventricular block	542 (48.3%)	87 (48.9%)	73 (43.7%)	101 (38.3%)	281 (54.8%)
Neurocardiogenic syncope	5 (0.4%)	1 (0.6%)	0 (0.0%)	1 (0.4%)	3 (0.6%)
Meets class I or II criteria for CRT	11 (1.0%)	1 (0.6%)	1 (0.6%)	0 (0.0%)	9 (1.8%)
Ablate and pace procedure	48 (4.3%)	7 (3.9%)	7 (4.2%)	11 (4.2%)	23 (4.5%)
Other	208 (18.5%)	34 (19.1%)	35 (21.0%)	64 (24.2%)	75 (14.6%)
**Associated diseases and risk factors**					
Hypertension	964 (85.9%)	150 (84.3%)	148 (88.6%)	238 (90.2%)	428 (83.4%)
Diabetes	435 (38.8%)	59 (33.1%)	58 (34.7%)	101 (38.3%)	217 (42.3%)
Renal disease	299 (26.6%)	44 (24.7%)	49 (29.3%)	64 (24.2%)	142 (27.7%)
Chronic pulmonary disease	229 (20.4%)	26 (14.6%)	28 (16.8%)	49 (18.6%)	126 (24.6%)
Pulmonary artery disease	102 (9.1%)	22 (12.4%)	10 (6.0%)	21 (8.0%)	49 (9.6%)
Other	391 (34.8%)	66 (37.1%)	64 (38.3%)	100 (37.9%)	161 (31.4%)
No associated diseases and risk factors	71 (6.3%)	14 (7.9%)	8 (4.8%)	10 (3.8%)	39 (7.6%)

ACE = angiotensin-converting enzyme; ARB = angiotensin receptor blocker; ARNi = angiotensin receptor–neprilysin inhibitor; BMI = body mass index; CRT = cardiac resynchronization therapy; HF = heart failure; HFpEF = heart failure with preserved ejection fraction; HFrEF = heart failure with reduced ejection fraction; LBBAP = left bundle branch area pacing; LVEF = left ventricular ejection fraction; PM = pacemaker; Q1 = first quartile; Q3 = third quartile; SD = standard deviation; SGLT2i = sodium-glucose cotransporter-2 inhibitor.

∗ Categories are not mutually exclusive.

Before the first recorded INGEVITY+ implant in the INSIGHT-LBBA registry, each of the 8 participating sites had varying degrees of familiarity with LBBAP implantation, with an overall higher level of experience using the lumen-less 3830 lead by Medtronic (5 of 8 sites with >50 implants) and less with other non-INGEVITY+ SDL leads by Biotronik or Abbott (1 site with >50 implants, Supplemental Table 1). Due to this variability in baseline experience with LBBAP, an additional subgroup analysis was performed comparing sites with extensive experience with lumenless LBBAP (sites 3, 4, 6, 7, and 8) and those with less experience with lumenless LBBAP (sites 1, 2, and 5), with overall similar baseline patient characteristics (Supplemental Table 2).

### Pacing outcomes

Overall LBBAP success rate was 95.3%, with moderate variability across different sites (Supplemental Figure 1), and a significant increase per higher implant order (adjusted OR, 1.10; 95% CI, 1.04–1.17, per every 10 successive LBBAP implants; *P* < .001; Table 3). Similarly, LBB capture success rate averaged 78.8%, with significant variability by implanting site (Supplemental Figure 2), and increased for higher implant order (adjusted OR, 1.04; 95% CI, 1.01–1.07, per every 10 successive implants; *P* = .006; Table 3). The use of multiple leads per implant was highest among implants 101+ (13.4% of patients; adjusted OR, 1.03; 95% CI, 1.00–1.05, per 10 consecutive implants; *P* = .03), but this was driven largely by a single site, and subgroup analysis with exclusion of the outlier site eliminated all significant differences across implant categories (Table 3). Pacing parameters at implant and 3 months were consistent as implant order increased (Table 3).

**Table 3:** Peri-procedural implant outcomes∗ in the overall cohort

Characteristic	Overall (N = 1374)	Implants 1–25 (N = 194)	Implants 26–50 (N = 173)	Implants 51–100 (N = 281)	Implants 101+ (N = 726)	Unadjusted		Adjusted	
						Estimate (95% CI)	*P*-value	Estimate (95% CI)	*P*-value
LBBAP success	1310 (95.3%)	184 (94.8%)	143 (82.7%)	273 (97.2%)	710 (97.8%)	1.07 (1.01–1.14)	.030	1.10 (1.04–1.17)	<.001
LBB capture success	1083 (78.8%)	157 (80.9%)	117 (67.6%)	222 (79.0%)	587 (80.9%)	1.02 (0.99–1.05)	.119	1.04 (1.01–1.07)	.006
More than 1 lead used per implant∗	132 (9.6%)	12 (6.2%)	11 (6.4%)	12 (4.3%)	97 (13.4%)	1.05 (1.03–1.08)	<.001	1.03 (1.00–1.05)	.030
Excluding outlier site	36/980 (3.7%)	5/169 (3.0%)	6/148 (4.1%)	4/231 (1.7%)	21/432 (4.9%)	1.02 (0.98–1.06)	.418	1.02 (0.99–1.04)	.183
Lead parameters at implant									
Sensing (mV)	12.3 (6.3)	12.3 (8.6)	12.4 (5.7)	11.7 (5.3)	12.6 (6.0)	0.02 (−0.00, 0.05)	.097	0.03 (−0.01, 0.06)	.115
Impedance (Ω)	852.7 (165)	840.9 (169)	844.6 (191)	859.1 (152)	855.1 (163)	0.36 (−1.19, 1.92)	.647	0.34 (−1.12, 1.80)	.647
Threshold (V at 0.4 ms)	0.8 (0.4)	0.8 (0.3)	0.9 (0.5)	0.8 (0.5)	0.8 (0.3)	−0.00 (−0.00, 0.00)	.138	−0.00 (−0.00, 0.00)	.156
Lead parameters at 3 Months†									
Sensing (mV)	16.2 (7.0)	16.0 (7.0)	15.1 (7.2)	16.7 (6.5)	16.4 (7.1)	0.01 (−0.02, 0.03)	.513	0.01 (−0.01, 0.04)	.241
Impedance (Ω)	699.9 (129)	702.4 (119)	688.7 (94.1)	701.7 (105)	701.3 (144)	0.56 (−0.32, 1.44)	.214	0.24 (−0.79, 1.27)	.650
Threshold (V at 0.4 ms)	1.0 (0.6)	1.0 (0.7)	0.9 (0.3)	0.9 (0.3)	1.0 (0.7)	−0.00 (−0.00, 0.00)	.475	0.00 (−0.00, 0.00)	.321

CI = confidence interval; LBB = lower bound bias; LBBAP = lower bound bias adjusted probability.

∗ Event rates and means (SD) presented for the categorized version of implant order. The continuous version of implant order was used for modeling purposes, and estimates are presented per a 10 unit increase in order.

† Lead parameter data supplemented with 6 months of data if 3 months were not available.

Notably, within the sensitivity analysis of only single- or dual-chamber pacemakers, the overall LBBAP success rate and LBB capture success rate were high at 95.6% and 80.7%, respectively. No significant associations were noted with implant volume in the unadjusted or adjusted models, albeit with a trend towards a higher LBB capture success rate with consecutive implants (adjusted OR, 1.11; 95% CI, 0.99–1.24; *P* = .076; Table 4). Furthermore, no significant differences were observed for number of leads used per implant or pacing parameters for later implants (Table 4). Procedural time analysis in the sensitivity cohort revealed stable total procedure time averaging 60.0 ± 37.1 minutes (Table 4). However, although fluoroscopy time remained minimal, averaging 10.0 ± 9.2 minutes overall, there was a statistically significant increase in fluoroscopy time with consecutive implants (mean difference [minute]: mean increase of 0.4 min [0.25, 0.56] per 10 consecutive implants; *P* < .001, Table 4) after the 100^th^ implant. There was no statistically significant difference in fluoroscopy time per 10 consecutive implants before the 100^th^ implant. Finally, all pacing parameters remained consistent with implant order both at implant and at 3-month follow-up (Table 4).

**Table 4:** Peri-procedural implant outcomes∗ in the sensitivity cohort

Characteristic	Overall (N = 1122)	Implants 1–25 (N = 178)	Implants 26–50 (N = 167)	Implants 51–100 (N = 264)	Implants 101+ (N = 513)	Unadjusted		Adjusted	
						Estimate (95% CI)	*P*-value	Estimate (95% CI)	*P*-value
LBBAP success	1073 (95.6%)	171 (96.1%)	140 (83.8%)	258 (97.7%)	504 (98.2%)	1.10 (0.99–1.23)	.085	1.11 (0.99–1.24)	.076
LBB capture success	906 (80.7%)	147 (82.6%)	116 (69.5%)	217 (82.2%)	426 (83.0%)	1.02 (0.97–1.08)	.417	1.02 (0.97–1.08)	.395
More than 1 lead used per implant	72 (6.4%)	11 (6.2%)	10 (6.0%)	11 (4.2%)	40 (7.8%)	1.00 (0.96–1.04)	.896	1.00 (0.96–1.04)	.945
Total procedure time (min)	60.0 (37.1)	71.0 (45.5)	64.7 (41.0)	50.7 (23.7)	59.4 (37.4)	−0.17 (-2.06, 1.71)	.857	−0.21 (−2.13, 1.70)	.826
Fluoroscopy time (min)†	10.0 (9.2)	11.1 (10.3)	9.1 (6.7)	8.0 (6.3)	11.4 (11.2)				
Implant numbers 0–100						−0.25 (−0.75, 0.25)	.334	−0.29 (−0.74, 0.15)	.199
Implant numbers >100						0.40 (0.25, 0.56)	<.001	0.41 (0.25, 0.57)	<.001
Lead parameters at implant									
Sensing (mV)	12.3 (6.3)	12.4 (8.7)	12.4 (5.8)	11.7 (5.3)	12.5 (6.0)	0.01 (−0.02, 0.05)	.345	0.02 (−0.02, 0.06)	.352
Impedance (Ω)	851.3 (157)	845.3 (169)	844.4 (190)	861.0 (152)	850.5 (142)	−0.19 (−2.15, 1.77)	.851	−0.38 (−2.33, 1.57)	.704
Threshold (V at 0.4 ms)	0.8 (0.4)	0.8 (0.3)	0.9 (0.5)	0.8 (0.5)	0.8 (0.3)	−0.00 (−0.00, 0.00)	.643	−0.00 (−0.00, 0.00)	0.669
Lead parameters at 3 Months‡									
Sensing (mV)	16.3 (6.9)	16.2 (7.0)	15.1 (7.3)	16.8 (6.5)	16.4 (6.9)	−0.00 (−0.02, 0.02)	.851	−0.00 (−0.02, 0.02)	.929
Impedance (Ω)	694.3 (103)	703.0 (120)	687.7 (93.9)	698.6 (105)	691.9 (100)	−0.13 (−1.73, 1.48)	.876	−0.09 (−1.72, 1.53)	.909
Threshold (V at 0.4 ms)	1.0 (0.6)	1.0 (0.7)	0.9 (0.3)	0.9 (0.3)	1.0 (0.7)	0.00 (−0.00, 0.01)	.197	0.00 (−0.00, 0.01)	.238

CI = confidence interval; LBB = lower bound bias; LBBAP = lower bound bias adjusted probability.

∗ Event rates and means (SD) presented for the categorized version of implant order. The continuous version of implant order was used for modeling purposes, and estimates are presented per a 10 unit increase in order.

† The relationship between implant number and fluoroscopy time was non-linear. Modeling was performed using 2 linear piecewise splines. Estimates are presented per a 10 unit increase in implant number, applicable to the implant numbers described.

‡ Lead parameter data supplemented with 6 months of data if 3 months is not available.

When comparing groups by prior lumenless lead experience (high- vs low-experienced sites), periprocedural characteristics including LBBAP success, procedure time, and fluoroscopy times were similar across these groups (Supplemental Table 3). Although the sites with less lumenless LBBAP experience reported higher use of more than 1 lead (11.8% vs 1.2%), the leads per implant were not evaluated in a model given the low event count for the high-experienced sites, so no statistical comparison could be made. Notably, there was a trend toward higher LBB capture success rate across sites with higher prior lumenless experience (89.7% vs 71.5%), although this was not statistically significant.

### Complications

Overall, LRC were low at 2.7% at 1 year and 3.7% at 2 years (Table 5, Figure 1). A majority of these complications involved lead dislodgement, with 2.2% by 1 year and 2.4% by 2 years (Table 5). Notably, the Kaplan–Meier curves indicated no events of lead dislodgement past 6 months among implants >26 (Figure 1). No significant differences by implant volume were observed in both unadjusted and adjusted analyses (adjusted HR for LRC, 1.01; 95% CI, 0.99–1.03; *P* = .40; adjusted HR for dislodgement, 1.01; 95% CI, 0.97–1.04; *P* = .70; Table 5). Within the sensitivity analysis cohort of non-CRT devices, complication rates were similarly low at 2.8% (any LRC) and 2.2% (lead dislodgement, Table 6), with all complications occurring in the first ∼8 months (Figure 2) and no further lead dislodgement past 6 months for all implant categories (Figure 2). Similarly, no significant associations were observed between implant order and complications. Lastly, when comparing sites grouped based on prior lumenless lead experience, no statistically significant differences in lead-related complications or lead dislodgements were noted between the high- and low-experienced sites (Supplemental Table 4).

**Table 5:** Post-procedural complications∗ in the overall cohort

Characteristic	Overall (N = 1374)	Implants 1–25 (N = 194)	Implants 26–50 (N = 173)	Implants 51–100 (N = 281)	Implants 101+ (N = 726)	Unadjusted		Adjusted†	
						Hazard Ratio (95% CI)	*P*-value	Hazard Ratio (95% CI)	*P*-value
Any lead-related complications									
Lead-related Complications						1.01 (0.99–1.03)	.499	1.01 (0.99–1.03)	.402
At 1 year	2.7% (33)	2.9% (5)	1.2% (2)	1.7% (4)	3.3% (22)				
At 2 years	3.7% (36)	4.3% (6)	1.2% (2)	1.7% (4)	5.3% (24)				
Lead dislodgement						1.00 (0.97–1.04)	.809	1.01 (0.97–1.04)	.698
At 1 Year	2.2% (27)	2.3% (4)	0.7% (1)	1.2% (3)	2.8% (19)				
At 2 Years	2.4% (28)	3.7% (5)	0.7% (1)	1.2% (3)	2.8% (19)				

CI = confidence interval.

∗ Kaplan–Meier event rates (event counts) presented for the categorized version of implant order. The continuous version of implant order was used for modeling purposes, and estimates are presented per a 10 unit increase in order.

† Adjustment variables include age, sex, heart failure etiology (if applicable), and device type.

**Figure 1:**
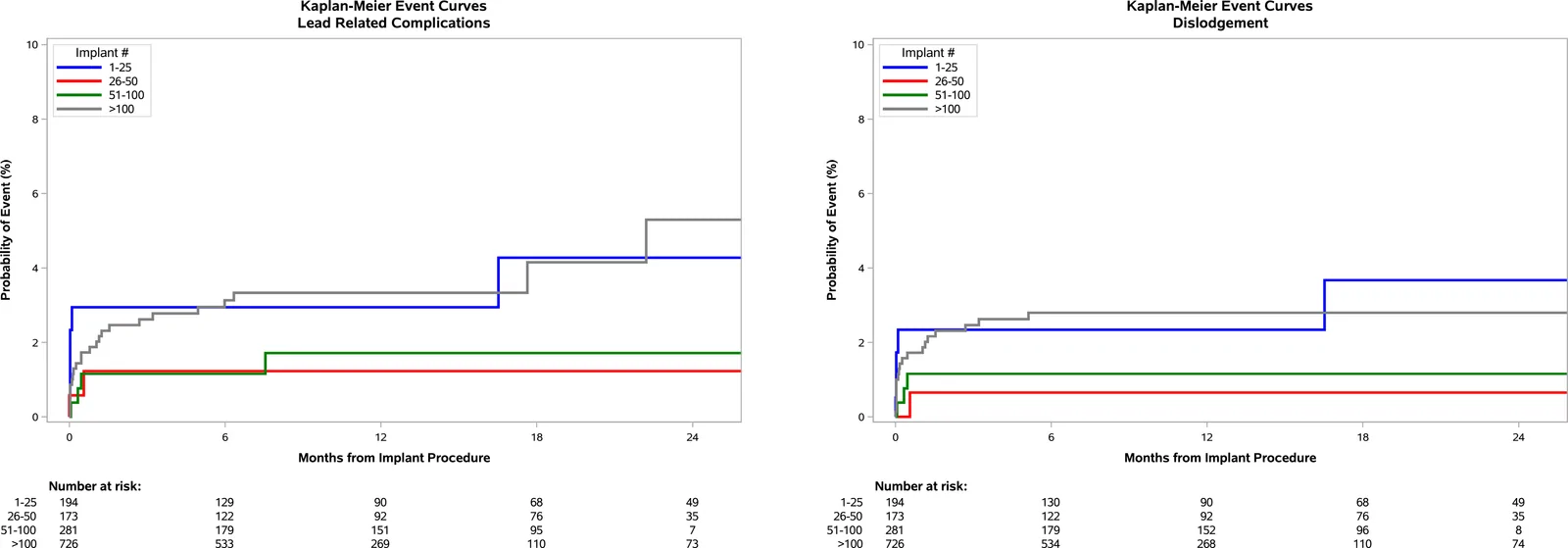
Kaplan–Meier plots for any lead-related complications (*left*) and lead dislodgements (*right*), by categorized implant order. Population: overall cohort.

**Table 6:** Post-procedural complications∗ in the sensitivity cohort

Characteristic	Overall (N = 1122)	Implants 1–25 (N = 178)	Implants 26–50 (N = 167)	Implants 51–100 (N = 264)	Implants 101+ (N = 513)	Unadjusted		Adjusted†	
						Hazard Ratio(95% CI)	*P*-value	Hazard Ratio (95% CI)	*P*-value
Any lead-related complications						1.02 (0.98–1.05)	.290	1.02 (0.98–1.05)	.381
At 1 year	2.8% (28)	3.1% (5)	1.3% (2)	1.8% (4)	3.6% (17)				
At 2 years	2.8% (28)	3.1% (5)	1.3% (2)	1.8% (4)	3.6% (17)				
Lead dislodgement						1.02 (0.97–1.07)	.509	1.02 (0.96–1.07)	.580
At 1 year	2.2% (23)	2.5% (4)	0.7% (1)	1.2% (3)	3.1% (15)				
At 2 years	2.2% (23)	2.5% (4)	0.7% (1)	1.2% (3)	3.1% (15)				

CI = confidence interval.

∗ Kaplan–Meier event rates (event counts) presented for the categorized version of implant order. The continuous version of implant order was used for modeling purposes, and estimates are presented per a 10-unit increase in order.

† Adjustment variables include age, sex, heart failure etiology, and device type.

**Figure 2:**
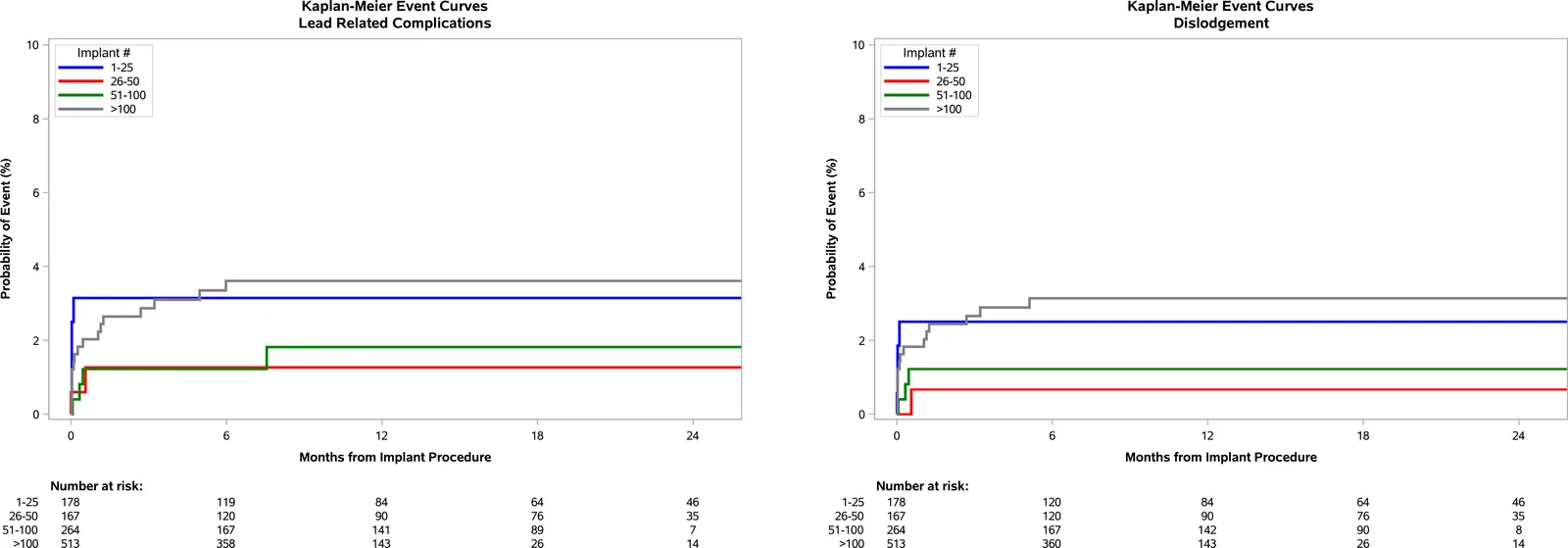
Kaplan–Meier plots for any lead-related complications (*left*) and lead dislodgements (*right*), by categorized implant order. Population: sensitivity cohort.

## Discussion

In this study, we performed a secondary analysis of the INSIGHT-LBBA multi-site registry assessing the safety and efficacy of INGEVITY+ leads for LBBAP. To our knowledge, this is the largest LBBAP study assessing procedural outcomes based on the implant order of the INGEVITY+ SDL. Overall, we identified that patients receiving later LBBAP implants were more likely to have HFrEF and meet criteria for CRT pacing. Furthermore, our data indicated that LBBAP success and LBB capture success rates continued to improve with consecutive cases, indicative of a learning curve for implant success. Finally, total procedural and fluoroscopy times, as well as the low rate of overall complications, exhibited no learning curve during LBBAP implantation with non-CRT systems.

Analysis of the baseline characteristics identified an overall homogeneous population of patients across different implant categories, with several notable exceptions for the later implant group (101+ implants). Specifically, patients in this cohort had 3–11× higher prevalence of idiopathic cardiomyopathy and HFrEF, and received either a CRT-P or CRT-D due to having met class I/II criteria for CRT. This may reflect a higher tendency for operators with more experience to implant LBBAP leads in patients that would normally receive a traditional CRT system with a CS lead, as limited evidence has supported the use of LBBAP instead of traditional CRT for patients with HFrEF.^6^, ^7^, ^8^ Given this significant discrepancy in baseline characteristics, a sensitivity analysis restricted to only single- or dual-chamber pacemakers was performed, with expected elimination of these baseline differences. Notably, the original cohort demonstrated a significant increase in both LBBAP success and LBB capture success with successive implants, with a 4%–10% increase in the odds of success for every 10 implants. Although the sensitivity analysis did not identify significant differences in LBB capture success for consecutive implants continuously, there was a trend towards a higher LBBAP success rate with later implants that barely missed statistical significance (OR, 1.11; 95% CI, 0.99–1.24; *P* = .076), likely due to a smaller cohort relative to the overall population. Linear assessment of fluoroscopy times suggested a 0.4-minute increase in fluoroscopy time for every 10 implants among implants >100—while statistically significant, this is likely of minimal clinical significance and may represent a tendency for operators to take on more challenging cases with increasing experience that may necessitate more fluoroscopy use. In contrast to the current analysis, the landmark MELOS study adopting LBBAP demonstrated longer learning curves, with the probability of LBBAP success slowly increasing until 270 cases, whereas fluoroscopy time for LBB capture decreased over the initial 110 cases.^2^ This mixed prospective and retrospective study encompassed 2533 patients across 14 European centers with implants between June 2018 and November 2021, with an average LBBAP success rate of 89.6%, and as such, likely reflects earlier adoption of LBBAP requiring a longer time to develop proficiency. Furthermore, 83.8% of implants used the Medtronic 3830 lumenless lead, with the remaining SDLs comprising the Solia (Biotronik) and Tendril (Abbott) leads, and only 9 cases using INGEVITY, making the results difficult to compare directly to those of INSIGHT-LBBA. Interestingly, analysis presented in supplemental data indicated a higher LBBAP success rate with SDLs relative to lumenless leads (92.9% vs 89.2%, *P* = .02), albeit at a cost of higher lead dislodgement rates (3.8% vs 1.1%, *P* < .001).^2^ Overall, LRC (2.6%) was similar to our current analysis. Subsequent analyses have failed to demonstrate significant differences in LBBAP success between SDLs and lumenless leads.^9^^,^^10^

More recently, the Conduction-System Pacing Italian Network Group (C-SING) collected prospective data on LBBAP procedures performed between January 2022 and December 2023 and identified that high-experience operators (>50 implants) had shorter fluoroscopy times (5.9 vs 9 minutes, *P* = .005) and a higher LBBAP success rate of 98.6% vs ∼92.5% for operators with ≤50 implants (*P* < .001).^11^ Although this partially corroborates our observed learning curves for LBBAP success, the C-SING study categorized implant experience by operator rather than by center, as was established in this secondary analysis. Unlike MELOS, a large fraction of patients (63.9%) in C-SING received an SDL, with the majority (51.1% total) using the Solia (Biotronik), and only 9.3% of all patients using the INGEVITY+. Similarly, several smaller single- and multi-center registries have shown high performance rates with evidence of a learning curve when implanting SDLs, although these studies exhibited a modest number of patients (50–353) and were strictly limited to the use of the Biotronik Solia lead.^12^, ^13^, ^14^ As such, our data corroborate the results of these studies, and more importantly, extend the high-performance dynamics and learning curves to the Ingevity+ lead by Boston Scientific among a broader patient population across 14 centers. However, this learning curve did not extend to fewer leads wasted in higher implant categories. Interestingly, 1 participating center used >1 lead per implant attempt significantly more frequently (14%) for implants 101+ relative to earlier implants, suggesting that increased experience may prompt operators to switch leads earlier due to improved knowledge of lead behavior. Furthermore, when comparing sites based on prior lumenless lead experience, those with limited experience on average used more than 1 lead more frequently relative to the higher-experienced sites, likely reflecting less familiarity with LBBAP implantation technique. This, however, was not analyzed statistically due to low event rates, and overall procedural characteristics and implant success were not different based on prior lumenless lead experience.

With respect to complications, both our primary and secondary analyses indicated an overall low rate of LRC (2.7%), consistent with other reports (2.6% in MELOS,^11^ and 3.4% in C-SING,^11^ 2%–5% across other studies,^12^, ^13^, ^14^, ^15^, ^16^ although with some smaller studies reporting ∼0% LRC complication rates^17^, ^18^, ^19^). Notably, the majority of the LRCs were due to lead dislodgement, which occurred early post-implant and was not observed after 5–8 months post-implant across all implant categories, with no evidence for a learning curve bias for increased early complications. The lead dislodgement rates trended towards a bimodal distribution, with the highest rates among the earliest and latest implanting categories, although not statistically significant. This may be a result of different factors, namely limited operator experience for the early groups, and increased tendency to take on tougher cases for the later groups, although the granularity of our data set does not provide details to support these speculations. Importantly, this low complication rate was independent of prior lumenless lead experience, indicating an excellent safety profile even for sites with less LBBAP experience.

### Limitations

The current study has multiple limitations worth noting. Given the deidentified and retrospective design of the INSIGHT-LBBA registry, LBB capture classification relied on investigator-reported criteria without central ECG adjudication, as the raw ECGs were not collected in the original registry. Furthermore, as LBB capture was determined based on a binary assessment of capture criteria, absolute values for V_6_-RWPT or V_6_–V_1_ interpeak interval were not available for assessing the degree of improvement with later LBBAP implants, as was suggested through the MELOS study.^2^ Due to significant inter-site heterogeneity, several outliers were noted that required partial subgroup analysis; however, due to the limited number of sites, extensive subgroup analysis was not performed to maintain adequate statistical power. Lastly, as the participating sites involved relatively experienced operators, results may not be translatable to the overall implanting population.

## Conclusion

This secondary analysis of the INSIGHT-LBBA registry identified that the success rate of both LBBAP and LBB capture was high and continued to improve with consecutive cases. Furthermore, overall complications remained low at 2.7% by 1 year, with no evidence of a learning curve bias for LRC, lead dislodgement, or procedural times. This study adds to the growing body of literature supporting the use of SDLs for LBBAP, and is the first to provide focused data on the relationship between site volume and procedural outcomes when using the INGEVITY+ lead for LBBAP.

## Disclosures

IYS has received research/grant support from the American Heart Association, National Institutes of Health, Boston Scientific, and Medtronic. ZL has received grant support from Boston Scientific and the Department of Veterans Affairs and serves as a consultant for Huxley Medical Inc., CardiaCures Inc., CathVision Inc, and Boston Scientific. GAU reports scientific advisory or speaking honoraria for Abbott, Biotronik, Boston Scientific, GE Medical, Medtronic, Philips, Rhythm Science, and Zoll Medical. SG reports consulting fees for Boston Scientific. KMT serves as a consultant and advisor for Abbott, Boston Scientific, and Medtronic. AP reports speaking and consulting honoraria from Boston Scientific. DJF has received research support from American Heart Association, Boston Scientific, Biosense Webster, Merit Medical, Medtronic, the National Institutes of Health, and Abbott; and consulting fees from Abbott, AtriCure, Microport, NI Medical, and Sanofi. HM, BA, NW, CAL, MYST, AL, JN, PWJ, and SBR all report no conflicts of interest.
