# Supplementary material for: Relationship between site procedural volumes and outcomes of left bundle branch area pacing using stylet-driven leads from the INSIGHT-LBBA Registry
Source: Heart Rhythm O2. 2026 Jul 20;7(10):1950–62. doi: 10.1016/j.hroo.2026.07.011 (PMC13700361; doi:10.1016/j.hroo.2026.07.011)
Supplement: Supplementary file 2 [file mmc2.pdf]

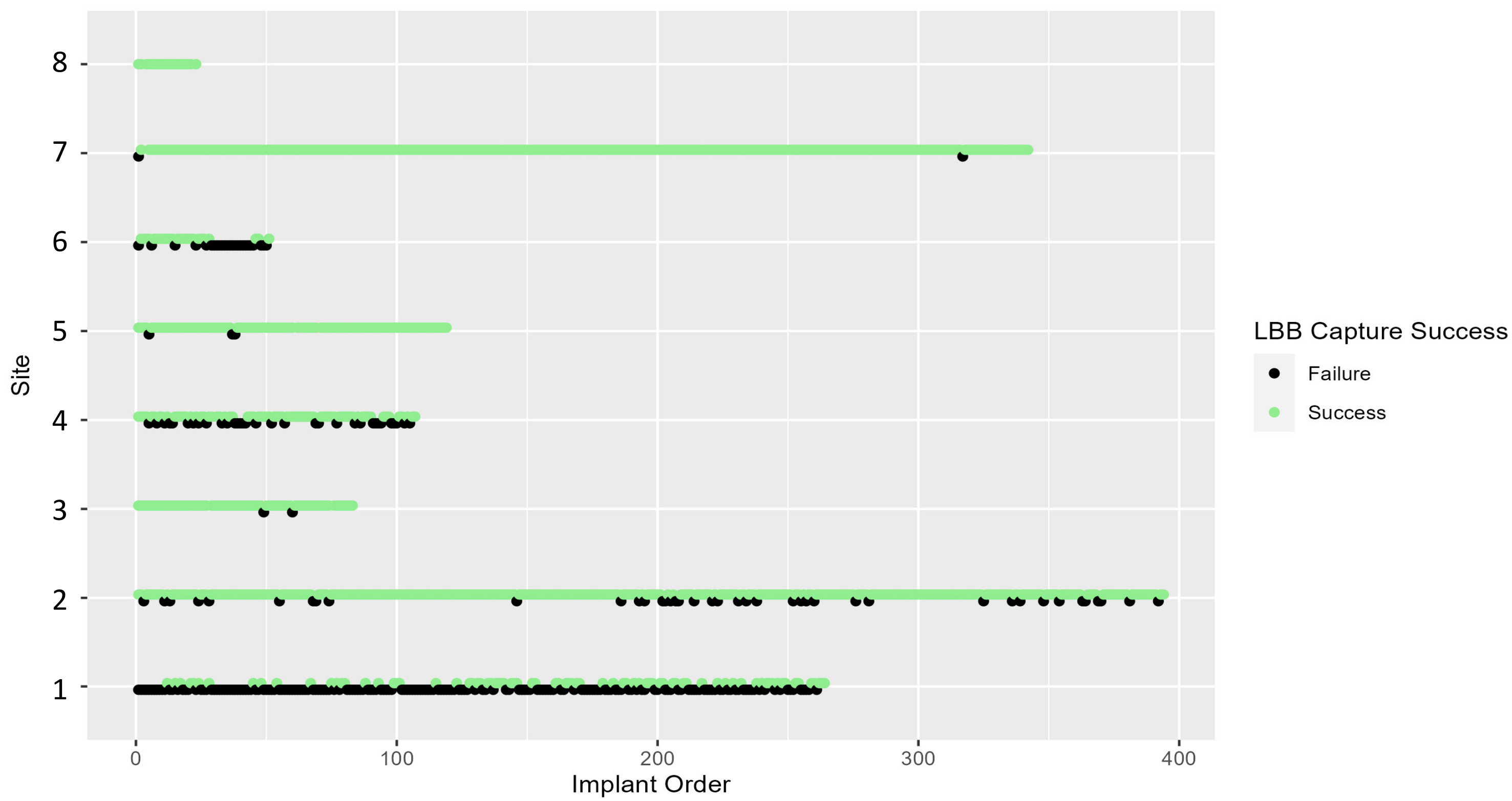

Supplemental Figure 2.
