# Supplementary material for: Relationship between site procedural volumes and outcomes of left bundle branch area pacing using stylet-driven leads from the INSIGHT-LBBA Registry
Source: Heart Rhythm O2. 2026 Jul 20;7(10):1950–62. doi: 10.1016/j.hroo.2026.07.011 (PMC13700361; doi:10.1016/j.hroo.2026.07.011)
Supplement: Supplementary file 3 [file mmc3.docx]

**Supplemental Figure legends**

**Supplemental** **Figure 1.** Dot plots of successive implants by site number illustrating either success or failure of LBBAP implant. LBBAP – left bundle branch area pacing.

**Supplemental** **Figure 2.** Dot plots of successive implants by site number illustrating either success or failure of capturing the LBB during LBBAP. LBB – left bundle branch; LBBAP – left bundle branch area pacing
