# Supplementary material for: Relationship between site procedural volumes and outcomes of left bundle branch area pacing using stylet-driven leads from the INSIGHT-LBBA Registry
Source: Heart Rhythm O2. 2026 Jul 20;7(10):1950–62. doi: 10.1016/j.hroo.2026.07.011 (PMC13700361; doi:10.1016/j.hroo.2026.07.011)
Supplement: Supplementary file 4 [file mmc4.docx]

**Supplemental Table 1: Pre-INSIGHT LBBAP implants by site**

|  | Site Number | | | | | | | |
| --- | --- | --- | --- | --- | --- | --- | --- | --- |
|  | 1 | 2 | 3 | 4 | 5 | 6 | 7 | 8 |
| Number of 3830 implants (Medtronic) | < 25 | < 25 | 50 - 99 | 50 - 99 | 25 - 49 | > 100 | 50 - 99 | > 100 |
| Number of non-Ingevity SDL implants (Biotronik/Abbott) | < 25 | < 25 | 25 - 49 | < 25 | < 25 | 25 - 49 | < 25 | > 100 |

**Supplemental Table 2: Baseline patient characteristics by site prior experience with Lumenless leads**

**Population: Sensitivity Cohort**

| **Characteristic** | **Overall (N=1,122)** | **Sites with Limited Experience (N=551)** | **Sites with More Experience (N=571)** |
| --- | --- | --- | --- |
| Number of sites |  | 3 | 5 |
| Age (yrs) |  |  |  |
| N | 1114 | 543 | 571 |
| Mean (SD) | 75.8 (10.0) | 75.4 (9.9) | 76.2 (10.0) |
| Median [Q1, Q3] | 76.9 (70.0, 82.0) | 76.0 (70.0, 82.0) | 77.0 (70.0, 83.0) |
| Male | 643 (57.3%) | 333 (60.4%) | 310 (54.3%) |
| Device type |  |  |  |
| Dual PM | 1073 (95.6%) | 526 (95.5%) | 547 (95.8%) |
| Single PM | 49 (4.4%) | 25 (4.5%) | 24 (4.2%) |
| Leads used per implant |  |  |  |
| 1 | 1050 (93.6%) | 486 (88.2%) | 564 (98.8%) |
| 2 | 59 (5.3%) | 52 (9.4%) | 7 (1.2%) |
| 3 | 13 (1.2%) | 13 (2.4%) | 0 (0.0%) |
| Race* |  |  |  |
| American Indian or Alaska Native | 8 (0.7%) | 6 (1.1%) | 2 (0.4%) |
| Asian | 75 (6.7%) | 56 (10.2%) | 19 (3.3%) |
| Black/African American | 118 (10.5%) | 43 (7.8%) | 75 (13.1%) |
| Native Hawaiian or Other Pacific Islander | 1 (0.1%) | 0 (0.0%) | 1 (0.2%) |
| White/Caucasian | 881 (78.5%) | 415 (75.3%) | 466 (81.6%) |
| Other/Unknown | 35 (3.1%) | 30 (5.4%) | 5 (0.9%) |
| Ethnicity |  |  |  |
| Hispanic or Latino | 33 (3.0%) | 18 (3.4%) | 15 (2.6%) |
| Not Hispanic or Latino | 1068 (96.7%) | 515 (96.4%) | 553 (97.0%) |
| Unknown | 3 (0.3%) | 1 (0.2%) | 2 (0.4%) |
| **Medications at Enrollment** |  |  |  |
| Ace Inhibitor | 251 (24.4%) | 128 (23.2%) | 123 (25.8%) |
| Angiotensin Receptor Blocker (ARB) | 238 (23.2%) | 157 (28.5%) | 81 (17.0%) |
| Angiotensin Receptor-Neprilysin Inhibitor (ARNi) | 5 (0.5%) | 4 (0.7%) | 1 (0.2%) |
| Flecainide | 20 (1.9%) | 10 (1.8%) | 10 (2.1%) |
| Propafenone | 0 (0.0%) | 0 (0.0%) | 0 (0.0%) |
| Other Antiarrhythmics | 105 (10.2%) | 57 (10.3%) | 48 (10.1%) |
| Anticoagulant | 432 (42.1%) | 231 (41.9%) | 201 (42.2%) |
| Diuretics | 406 (39.5%) | 241 (43.7%) | 165 (34.7%) |
| Beta Blockers | 436 (42.5%) | 252 (45.7%) | 184 (38.7%) |
| Sodium-glucose Cotransporter-2 (SGLT2i) | 56 (5.5%) | 34 (6.2%) | 22 (4.6%) |
| Aldosterone Antagonists | 56 (5.5%) | 47 (8.5%) | 9 (1.9%) |
| Calcium Channel Blockers | 334 (32.5%) | 179 (32.5%) | 155 (32.6%) |
| Clinical heart failure diagnosis | 414 (36.9%) | 216 (39.2%) | 198 (34.7%) |
| Ischemic heart disease | 85 (7.6%) | 50 (9.1%) | 35 (6.1%) |
| Idiopathic cardiomyopathy | 101 (9.0%) | 61 (11.1%) | 40 (7.0%) |
| Valvular heart disease | 87 (7.8%) | 64 (11.6%) | 23 (4.0%) |
| Hypertrophic cardiomyopathy | 11 (1.0%) | 9 (1.6%) | 2 (0.4%) |
| Congenital heart disease | 3 (0.3%) | 1 (0.2%) | 2 (0.4%) |
| Left ventricular hypertrophy | 62 (5.5%) | 57 (10.3%) | 5 (0.9%) |
| Infiltrative cardiomyopathy | 7 (0.6%) | 3 (0.5%) | 4 (0.7%) |
| No disease (age related) | 89 (7.9%) | 12 (2.2%) | 77 (13.5%) |
| HF Type |  |  |  |
| Heart Failure with Preserved Ejection Fraction (HFpEF) | 309 (27.5%) | 159 (28.9%) | 150 (26.3%) |
| Heart Failure with Reduced Ejection Fraction (HFrEF) | 96 (8.6%) | 56 (10.2%) | 40 (7.0%) |
|  |  |  |  |
| **Cardiac Assessment** |  |  |  |
| QRS width pre-LBBAP implant (intrinsic) |  |  |  |
| N | 1057 | 531 | 526 |
| Mean (SD) | 116.2 (29.2) | 117.5 (28.7) | 115.0 (29.7) |
| Median [Q1, Q3] | 111 (92.0, 140) | 114 (92.0, 142) | 109 (92.0, 138) |
| QRS width pre-LBBAP implant (paced) |  |  |  |
| N | 29 | 20 | 9 |
| Mean (SD) | 156.3 (28.2) | 162.2 (28.8) | 143.3 (23.1) |
| Median [Q1, Q3] | 160 (145, 165) | 160 (148, 172) | 145 (134, 160) |
| QRS width post-LBBAP implant (paced) |  |  |  |
| N | 617 | 371 | 246 |
| Mean (SD) | 123.0 (25.1) | 119.3 (15.7) | 128.6 (34.0) |
| Median [Q1, Q3] | 122 (108, 134) | 118 (108, 128) | 129 (110, 143) |
| Cardiac surgeries within 30 days of implant | 94 (8.4%) | 89 (16.2%) | 5 (0.9%) |
| LVEF |  |  |  |
| N | 1028 | 536 | 492 |
| Mean (SD) | 57.7 (7.7) | 57.8 (7.7) | 57.6 (7.7) |
| Median [Q1, Q3] | 57.0 (55.0, 63.0) | 55.0 (55.0, 63.0) | 57.0 (55.0, 63.0) |
| Septal wall thickness (mm) |  |  |  |
| N | 896 | 488 | 408 |
| Mean (SD) | 11.6 (2.7) | 11.6 (2.7) | 11.6 (2.8) |
| Median [Q1, Q3] | 11.0 (10.0, 13.0) | 11.0 (10.0, 13.0) | 11.0 (9.7, 13.0) |
| BMI |  |  |  |
| N | 1091 | 551 | 540 |
| Mean (SD) | 29.3 (6.6) | 29.1 (6.4) | 29.6 (6.7) |
| Median [Q1, Q3] | 28.3 (24.6, 33.0) | 28.1 (24.2, 32.5) | 28.5 (24.8, 33.6) |
| Pacing indications* |  |  |  |
| Sinus node dysfunction | 448 (39.9%) | 211 (38.3%) | 237 (41.5%) |
| Atrioventricular block | 542 (48.3%) | 331 (60.1%) | 211 (37.0%) |
| Neurocardiogenic syncope | 5 (0.4%) | 4 (0.7%) | 1 (0.2%) |
| Meets class I or II criteria for CRT | 11 (1.0%) | 11 (2.0%) | 0 (0.0%) |
| Ablate and pace procedure | 48 (4.3%) | 47 (8.5%) | 1 (0.2%) |
| Other | 208 (18.5%) | 31 (5.6%) | 177 (31.0%) |
| **Associated diseases and risk factors** |  |  |  |
| Hypertension | 964 (85.9%) | 484 (87.8%) | 480 (84.1%) |
| Diabetes | 435 (38.8%) | 209 (37.9%) | 226 (39.6%) |
| Renal disease | 299 (26.6%) | 169 (30.7%) | 130 (22.8%) |
| Chronic pulmonary disease | 229 (20.4%) | 162 (29.4%) | 67 (11.7%) |
| Peripheral arterial disease | 102 (9.1%) | 73 (13.2%) | 29 (5.1%) |
| Other | 391 (34.8%) | 251 (45.6%) | 140 (24.5%) |
| No associated diseases and risk factors | 71 (6.3%) | 28 (5.1%) | 43 (7.5%) |

*Categories are not mutually exclusive.

**Supplemental Table 3: Peri-procedural Implant Outcomes**

**Population: Sensitivity Cohort**

|  | | | **Unadjusted** | | **Adjusted^2^** | |
| --- | --- | --- | --- | --- | --- | --- |
| **Outcome** | **Sites with Limited Experience^1^ (N=551)** | **Sites with More Experience^1^ (N=571)** | **Estimate (95% CI)** | **P-value** | **Estimate (95% CI)** | **P-value** |
| LBBAP success | 527 (95.6%) | 546 (95.6%) | 1.01 (0.10 - 9.69) | 0.996 | 1.13 (0.12 - 11.0) | 0.913 |
| LBB capture success | 394 (71.5%) | 512 (89.7%) | 0.29 (0.03 - 2.96) | 0.295 | 0.28 (0.03 - 2.87) | 0.281 |
| More than 1 lead used per implant^3^ | 65 (11.8%) | 7 (1.2%) | - |  | - |  |
|  |  |  |  |  |  |  |
| Total procedure time (min) | 54.8 (31.9) | 72.7 (45.3) | -17.9 (-60.7, 24.93) | 0.413 | -20.6 (-63.0, 21.76) | 0.341 |
| Fluoroscopy time (min) | 10.1 (9.4) | 9.6 (8.8) | 0.52 (-5.10, 6.13) | 0.857 | -0.09 (-5.43, 5.25) | 0.974 |

^1^Event rates and means (SD) presented.

^2^Adjustment variables included age, sex and heart failure etiology (if applicable).

^3^The leads per implant outcome was not evaluated in a model given the low event count for high experience sites.

**Supplemental Table 4: Post-procedural Complications**

**Population: Sensitivity Cohort**

|  | | | **Unadjusted** | | **Adjusted^2^** | |
| --- | --- | --- | --- | --- | --- | --- |
| **Outcome** | **Sites with Limited Experience^1^ (N=551)** | **Sites with More Experience^1^ (N=571)** | **Hazard Ratio (95% CI)** | **P-value** | **Hazard Ratio (95% CI)** | **P-value** |
| Any lead-related complications | 2.6% (13) | 3.0% (15) | 0.92 (0.41 - 2.10) | 0.850 | 0.94 (0.44 - 2.00) | 0.878 |
| Lead dislodgement | 2.4% (12) | 2.1% (11) | 1.15 (0.40 - 3.24) | 0.797 | 1.16 (0.45 - 3.01) | 0.758 |

^1^Kaplan-Meier event rates (event counts) are presented at 2 years. The event rates at 1 and 2 years are the same.

^2^Adjustment variables include age, sex and heart failure etiology (if applicable).
